# Antipsychotic Use and Mortality in Persons with Alcohol-Related Dementia or Wernicke–Korsakoff Syndrome: A Nationwide Register Study in Finland

**DOI:** 10.3390/jcm12134263

**Published:** 2023-06-25

**Authors:** Anniina Palm, Tiina Talaslahti, Risto Vataja, Milena Ginters, Hannu Kautiainen, Henrik Elonheimo, Jaana Suvisaari, Nina Lindberg, Hannu Koponen

**Affiliations:** 1 Department of Psychiatry, Helsinki University Hospital, 00029 Helsinki, Finland; 2 University of Helsinki, 00014 Helsinki, Finland; 3 Primary Health Care Unit, Kuopio University Hospital, 70210 Kuopio, Finland; 4 Folkhälsan Research Center, 00250 Helsinki, Finland; 5 Finnish Institute for Health and Welfare, 00271 Helsinki, Finland

**Keywords:** Wernicke–Korsakoff syndrome, alcohol-related dementia, antipsychotics, mortality

## Abstract

Background: Research on the use of psychotropic drugs in people with alcohol-related neurocognitive disorders is virtually nonexistent. We examined the prevalence of antipsychotic drug use and its effect on mortality among patients with Wernicke–Korsakoff syndrome (WKS) or alcohol-related dementia (ARD). Methods: In this nationwide register study, we collected data on the medication use and mortality of all persons aged ≥40 diagnosed with WKS (n = 1149) or ARD (n = 2432) between 1998 and 2015 in Finland. We calculated the prevalence of antipsychotic use within one year of diagnosis and the adjusted cumulative mortality of antipsychotic users versus non-users in relation to the age-, sex-, and calendar year-matched general population. Results: Of the WKS and ARD patients, 35.9% and 38.5%, respectively, purchased one or more antipsychotic drugs in the year following diagnosis. The adjusted cumulative mortality of the antipsychotic users was significantly lower than that of non-users in both the WKS and ARD groups, where the adjusted hazard ratios (95% CI) were 0.85 (0.72–0.99) and 0.73 (0.65–0.81), respectively. WKS and ARD patients using antipsychotics were less likely to die of alcohol-related causes than antipsychotic non-users, but the difference was significant only in the ARD group. Conclusions: This population-based study shows that antipsychotic use is common in patients with WKS or ARD. In contrast to other dementia studies, our results indicate that the mortality of antipsychotic users is significantly lower than that of non-users. The lower mortality could be explained by decreased alcohol use and better healthcare coverage in antipsychotic users.

## 1. Introduction

Alcohol-related neurocognitive disorders are relatively common causes of dementia, especially in younger age groups. These disorders constitute approximately 10% of etiologies in early-onset dementia [1] and around 0.8–1.3% of late-onset dementia cases [2,3].

Alcohol-related cognitive impairment is an umbrella term that comprises mainly two syndromes: Wernicke–Korsakoff syndrome (WKS) and alcohol-related dementia (ARD) [4]. WKS is a neuropsychiatric disorder caused by thiamine (vitamin B1) deficiency. It manifests first as acute Wernicke encephalopathy (WE) and is clinically characterized by a classic triad of symptoms: mental status changes, oculomotor symptoms, and cerebellar dysfunction, with a minority of patients expressing all three [5]. In Western studies, up to 90% of WE cases are related to alcohol use. Untreated alcohol-related WE leads to a chronic amnestic condition known as Korsakoff syndrome in 56–84% of patients [5,6].

Similarly, ARD is a syndrome of severe cognitive impairment resulting from substantial long-term alcohol abuse. The etiopathology of ARD is not well-established; the dementia might be caused by direct ethanol neurotoxicity or by thiamine deficiency (i.e., ARD being a variant of WKS), or it could be multifactorial [7]. The cognitive profile of ARD is characterized by a more global cognitive decline than seen in WKS [4]. Although not widely in clinical use, the diagnostic criteria of Oslin et al. (1998) suggest that ARD requires a five-year period of consuming at least 35 standard drinks per week for men or 28 for women to develop.

Persons with dementia commonly suffer from neuropsychiatric symptoms (NPS) such as agitation, anxiety, apathy, depression, and psychotic symptoms. Up to 80–90% of dementia patients experience NPS during the course of the disease [8,9]. As in other forms of dementia, NPS are also prevalent in alcohol-related neurocognitive disorders; a recent review study concluded that psychotic symptoms affected a median of 10% (range 0–87%) of patients, while agitation and aggression were seen in 27% (range 10–54%) [10].

NPS in dementia are frequently treated with antipsychotic medication, even though these drugs show only modest benefit and may have serious adverse effects [11]. Older patients with dementia who receive antipsychotics have an increased risk of mortality [12,13], the risk being higher with haloperidol than with atypical antipsychotics [12]. Antipsychotic use for NPS is also associated with a higher risk of cerebrovascular adverse events, sedation, and gait abnormalities [14].

Research on the use of psychotropic drugs among people with alcohol-related neurocognitive disorders is almost nonexistent. We are only aware of two reports from Dutch nursing homes that have documented the use of antipsychotic medication in this patient group. In their cross-sectional studies of institutionalized patients with Korsakoff syndrome or other alcohol-related neurocognitive disorders [15,16], the authors reported that 67–71% of patients used one or more psychotropic drugs, most commonly antipsychotics (47–48%), antidepressants (38–40%), and benzodiazepines (34–36%). Of these nursing home residents, 59% had at least one comorbid psychiatric condition, the most frequent ones being mood disorders (31–32%), psychotic disorders (18–22%), and personality disorders (12–15%).

To our knowledge, no earlier research exists on the use of antipsychotic drugs in non-institutionalized people with WKS or ARD, nor on the impact of antipsychotics on the mortality of these patients. Therefore, we decided to conduct this nationwide register study to determine the prevalence of antipsychotic use after receiving a diagnosis of WKS or ARD and to investigate the effect of antipsychotic use on the mortality and causes of death of these patients.

## 2. Methods

### 2.1. Register Data

This observational study used retrospective data obtained from the Finnish Hospital Discharge Register (FHDR, currently known as the Care Register for Health Care). The FHDR is a comprehensive nationwide register that has been in operation since 1969, collecting mandatory data as per Finnish law [17]. The FHDR contains information on inpatient service and outpatient visits in specialized healthcare and primary care outpatient visits. Inpatient data are accumulated from public hospitals, municipal health center wards, and other (private, military, and prison) hospitals. Information on outpatient visits in specialized healthcare is limited to public services. Since 2011, the FHDR has also included data on all outpatient visits to public primary care. The register data contain the main diagnoses and subdiagnoses of the hospital stay, information about the procedures and interventions, and patient characteristics such as age, sex, and residence status. Diagnoses in the FHDR have been recorded using the ICD-10 system since 1996. The register has a high coverage rate, exceeding 95% in recent years [17,18], and approximately 1.7 million individuals’ data are reported to the FHDR annually [19]. Data on the population, mortality, and causes of death were obtained from Statistics Finland, which registers official statistics on deaths (including causes of death) of all persons permanently domiciled in Finland.

Data on medication were acquired from the prescription register of the Social Insurance Institution of Finland, which holds nationwide records of every reimbursed drug purchase by Finnish residents. The national health insurance in Finland provides coverage for all residents, ensuring that prescription medications, including antipsychotics, are reimbursed. As a result, the national prescription register maintains a comprehensive record of medication usage, offering nearly complete coverage of the entire population. Data were linked by the personal identity code issued to every person registered in Finland.

### 2.2. Study Population

By integrating data from numerous population-based nationwide registers, we constructed a dataset that encompassed virtually the entire population of Finland (around 5.5 million inhabitants). We extracted from the FHDR all Finnish residents who had received a diagnosis of WKS (n = 1149, 841 men and 308 women) or ARD (n = 2432, 1892 men and 540 women) between 1998 and 2015 and who were aged ≥40 years at diagnosis. The lower age limit was set since no persons under 40 with a diagnosis of WKS or ARD were found in the register data. We then extracted all drug purchases with the Anatomical Therapeutic Chemical (ATC) Classification System code N05A (antipsychotics, excluding lithium) made by the study population. Patients were considered users of antipsychotics if they had any purchases of antipsychotic medications within one year after the diagnosis of WKS or ARD. The patients were followed from 1998 to 2015 or until death. Data on mortality and the causes of death were collected from death certificates until the end of 2018.

The classification of diagnoses was based on the 10th revision of the World Health Organization International Classification of Diseases (ICD-10) [20]. The study population included all cases with ICD-10 diagnoses F10.6 (Korsakoff syndrome or alcohol-induced amnesic syndrome) for WKS and F10.73 (alcohol-related dementia or alcoholic dementia) for ARD. In analyzing the causes of death, alcohol-related diseases included all diseases caused by alcohol (ICD-10 categories F10, G31.2, G40.51, G62.1, G72.1, I42.6, K29.2, K70, K85.2, K86.0, O35.4, P04.3, and Q86.0).

### 2.3. Data Analysis

The baseline characteristics of the study population stratified by use of antipsychotic medication are presented using descriptive statistics: continuous variables are summarized as the mean and standard deviation (%) and categorical variables as the frequency and percentage (%). Cox proportional hazards regression was used to estimate the adjusted hazard ratios (HRs) and their 95% confidence intervals (CIs). Adjusted survival curves were based on a stratified Cox model using baseline age and sex as covariates. The Cox proportional hazards (PH) assumption was tested graphically (using a plot of the log cumulative hazard, where the logarithm of time is plotted against the estimated log cumulative hazard calculated as ln[−ln (S(t))]) and by the use of a statistical test based on the distribution of Schoenfeld residuals. A Fine and Gray competing risks regression model to calculate subhazard ratios (sHRs) regression was used to analyze the risk of death due to different causes of death (competing risk). For each cause of death, the rest of the causes were considered as competing risks. The ratio of the observed to expected number of deaths, i.e., the standardized mortality ratio (SMR) for all-cause deaths, was calculated using subject-years methods with 95% CIs. The expected number of deaths was calculated on the basis of sex-, age-, and calendar-period-specific mortality rates in the Finnish population (Official Statistics of Finland). Hommel’s adjustment was applied to correct levels of significance for multiple testing, if appropriate. The Stata 17.0 (StataCorp LP; College Station, TX, USA) statistical package was used for the analysis.

## 3. Results

Table 1 presents an overview of the study population stratified by use of antipsychotic medication. In the year following diagnosis, 35.9% of WKS patients and 38.5% of ARD patients purchased one or more antipsychotic drugs. The antipsychotic user and non-user groups were similar in sex ratio; the age at diagnosis was similar in the WKS group but differed slightly in the ARD group.

**Figure 1:**
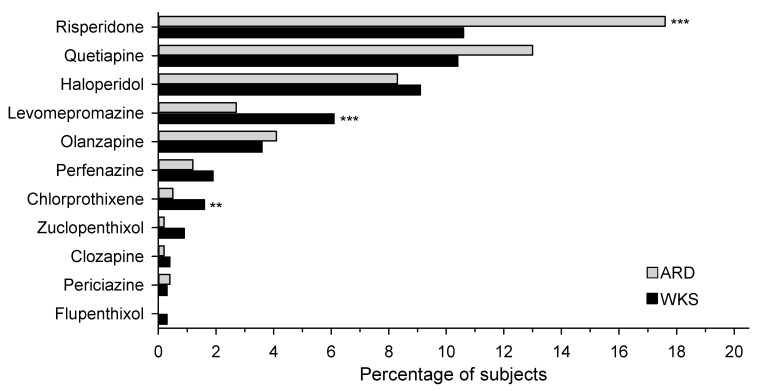
Antipsychotic purchases within one year of diagnosis. Hommel’s multiple comparison procedure was used to correct significance levels for post hoc testing; ** *p* < 0.01, *** *p* < 0.001. ARD: alcohol-related dementia; WKS: Wernicke–Korsakoff syndrome.

The most commonly used antipsychotics were risperidone, quetiapine, and haloperidol (Figure 1). The number of patients in the WKS and ARD groups who used risperidone was 10.6% and 17.6%, quetiapine 10.4% and 13.0%, and haloperidol 9.1% and 8.3%, respectively. Among patients with WKS or ARD, 63.9% and 76.5% of the purchased antipsychotic drugs were atypical antipsychotics, respectively, while the rest were first-generation antipsychotics.

**Figure 2:**
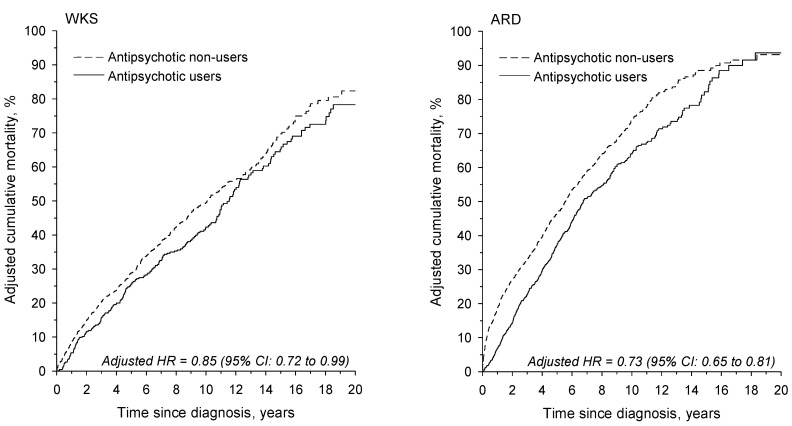
Age- and sex-adjusted cumulative mortality in WKS and ARD patients. ARD: alcohol-related dementia; HR: hazard ratio; WKS: Wernicke–Korsakoff syndrome.

The age- and sex-adjusted cumulative mortality of antipsychotic users was significantly lower than that of non-users, as shown in Figure 2. The adjusted HR for antipsychotic users was 0.85 (95% CI: 0.72–0.99) in the WKS group and 0.73 (95% CI: 0.65–0.81) in the ARD group relative to antipsychotic non-users.

**Figure 3:**
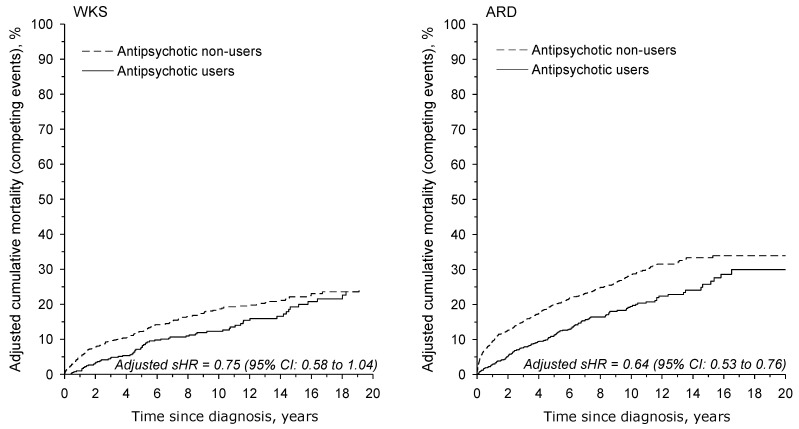
Age- and sex-adjusted cumulative mortality due to alcohol-related causes in WKS and ARD patients. We calculated the cumulative mortality in the presence of competing events, where the rest of the causes of death were considered as competing risks. ARD: alcohol-related dementia; sHR: subhazard ratio; WKS: Wernicke–Korsakoff syndrome.

The age- and sex-adjusted mortality due to alcohol-related causes was also significantly lower in antipsychotic users versus non-users. The adjusted sHR for antipsychotic users was 0.78 (95% CI: 0.58–1.04) in the WKS group and 0.64 (95% CI: 0.53–0.76) in the ARD group relative to antipsychotic non-users (Figure 3).

**Figure 4:**
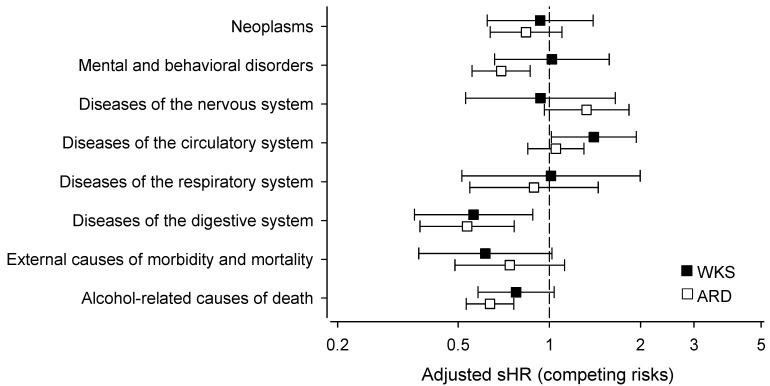
Age- and sex-adjusted subhazard ratio (sHR) of WKS and ARD patients using antipsychotics compared with antipsychotic non-users, stratified by causes of death (ICD-10 diagnosis groups). The value for antipsychotic non-users is represented by the dotted line, i.e., 1. For calculating sHRs, we used the competing risks regression model, where the rest of the causes of death were considered as competing risks. ARD: alcohol-related dementia; sHR: subhazard ratio; WKS: Wernicke–Korsakoff syndrome.

Figure 4 shows the adjusted mortality of the antipsychotic users compared with the antipsychotic non-users (represented by the dotted line, i.e., 1). The sHRs were stratified by the causes of death using ICD-10 categories. The mortality of antipsychotic users due to diseases of the digestive system was lower in both the WKS patients (sHR: 0.56, 95% CI: 0.36–0.88) and ARD patients (sHR: 0.54, 95% CI: 0.37–0.77). The ARD patients using antipsychotics were also significantly less likely to die of mental and behavioral disorders (sHR: 0.69, 95% CI: 0.56–0.87) than the antipsychotic non-users. The antipsychotic users with ARD were more likely to die of diseases of the circulatory system (sHR: 1.40, 95% CI: 1.01–1.94) than the antipsychotic non-users. Although not statistically significant, antipsychotics users had a tendency towards a lower mortality of external causes of death—a category that includes deaths caused by, for instance, intoxication, accidents, assault, and suicide. The mortality due to alcohol-related causes was lower in both the WKS and ARD patients using antipsychotics, as recounted in the previous paragraph and shown in Figure 2, although the difference was significant in the ARD group only.

## 4. Discussion

To our knowledge, this is the first population-based study that describes the use of antipsychotic drugs among people with WKS or ARD. We show that antipsychotic purchases were very common in this patient group. In contrast to other dementia studies, our results indicate that the age- and sex-adjusted cumulative mortality of antipsychotic users with a diagnosis of WKS or ARD was significantly lower than that of non-users. When assessing causes of death, both the WKS and ARD patients who had purchased antipsychotics were less likely to die of alcohol-related causes than antipsychotic non-users, but the difference was significant only in the ARD group.

Antipsychotic use was frequent among the WKS and ARD patients in our study. While 20% of people with Alzheimer’s disease had purchased an antipsychotic within a year after diagnosis in Finland [21], the corresponding figure for the WKS and ARD patients in our data (36% in the WKS group and 39% in the ARD group) was almost twice as high. Compared with the non-institutionalized persons in our study, Gerridzen and Goossensen [15] reported that nursing home patients with alcohol-related neurocognitive disorders were even more likely to use antipsychotics; in total, 48% of such patients used antipsychotic medication. In their study, a large number of patients did not have a diagnosis of a comorbid psychotic disorder, implying that antipsychotics were used to treat NPS in the long-term care setting. Presumably, patients who ended up in nursing homes would show more severe cognitive deficits and more NPS than non-institutionalized patients, explaining the difference in the prevalence of antipsychotic use.

Although our data did not include clinical indications for antipsychotic medication, it is likely that these drugs were often used to treat NPS—a relatively common off-label indication for antipsychotic use. In nursing home patients with alcohol-related neurocognitive disorders, antipsychotics were used both for psychotic disorders (32%), behavioral symptoms (30%), or a combination of both (10%); in particular, irritability and agitation/aggression were associated with antipsychotic prescriptions [22]. People with WKS or ARD tend to be younger than other dementia patients and physically more active since they are less often impaired in daily activities [16]. However, in addition to memory deficits, these patients display severe difficulties in executive functions [23] and self-awareness of their condition [16]. They are prone to delusions, hallucinations, agitation, aggression, and disinhibition [10,16], symptoms often regarded as difficult to treat and associated with caregiver distress [24].

## 5. Mortality

Use of antipsychotic drugs in older people and persons with dementia has been associated with serious adverse drug effects, including elevated mortality risk [12]. In contrast, our data showed decreased all-cause mortality among antipsychotic users relative to non-users in people diagnosed with WKS or ARD. Furthermore, patients using antipsychotics were less likely to die of alcohol-related causes than antipsychotic non-users, although the difference was significant only in the ARD group. Pertaining to mortality, we have shown earlier that WKS and ARD are disorders with a poor prognosis; compared with the general population, patients with WKS or ARD have a fivefold mortality, and they are considerably more likely to die of alcohol-related causes [25].

Although the mechanisms underlying decreased mortality cannot be ascertained from our data, reduced alcohol use among antipsychotic users could explain the phenomenon. The effect of antipsychotic drugs is mediated through antagonism of dopamine, a neurotransmitter involved in the rewarding effects of drugs and alcohol. In theory, antipsychotic drugs could be useful in improving the symptoms or even outcomes of substance use disorders [26]. Several studies have indicated that atypical antipsychotics may decrease alcohol use in patients with alcohol use disorder but possibly only in some subtypes of alcoholics [26].

The efficacy of antipsychotic treatment has also been studied in people with a dual diagnosis of substance use disorder and psychosis. There is limited evidence that clozapine and other atypical antipsychotics, especially long-acting injectable formulations, may lead to improvements in alcohol use in dual diagnosis patients. First-generation antipsychotics do not appear to be useful in treating alcohol use disorder [26,27,28].

Lower levels of alcohol consumption among antipsychotic users could also explain other observations in our data. Compared with non-users, antipsychotic users were less likely to die of gastrointestinal diseases, of which the most common cause of death was alcoholic liver disease. Similarly, ARD patients using antipsychotics were less likely to die of mental and behavioral disorders; the most common causes of death in this group were disorders due to psychoactive substance use. Antipsychotics may also have an effect on impulsive behavior; in our data, patients using antipsychotics had a tendency towards lower mortality from unnatural causes of death (i.e., intoxication, accidents, assault, and suicide), although the difference did not reach statistical significance.

Furthermore, our study population was considerably younger (mean age at diagnosis 57 years) than in other dementia studies. For instance, the mean age of dementia patients in earlier meta-analyses assessing antipsychotic use and mortality was around 81 years [11,13]. Possibly, the increased mortality risk of antipsychotics is exacerbated in older and frailer patients.

Antipsychotic drugs are known to have cardiac side effects, such as arrythmias and metabolic changes [29]. In older patients with dementia, off-label use of risperidone and olanzapine was associated with a higher risk of cardiovascular events [30]. Indeed, cardiovascular adverse effects may explain our finding that in the WKS group antipsychotic users were more likely to die of cardiovascular causes than antipsychotic non-users.

Since register studies cannot establish causality, the possibility exists that patients who continued to abuse alcohol and had a higher mortality from alcohol-related causes were also less likely to seek healthcare or to adhere to treatment. This might lead to them not receiving drug prescriptions or not purchasing the prescribed medication. Thus, drug prescriptions might serve as indicators of received healthcare, which in turn leads to better mortality outcomes.

## 6. Strengths and Limitations

The main strength of our study was the comprehensive population-based register data on these patient groups, who may be difficult to reach otherwise for clinical research. This nationwide study included all patients in Finland with a clinical diagnosis of WKS or ARD and all antipsychotic drug purchases made by these patients within one year after diagnosis. The followup time was long, from 1998 to 2015. The quality and reliability of Finnish healthcare registers are deemed high [17]. Data from the prescription register include actual medication purchases instead of merely prescriptions and are free from reporting or recall bias. While the medication use itself could not be verified, an earlier Finnish study reported a substantial correlation between pharmacy data and self-reported antipsychotic use [31].

This study had a number of limitations, partly inherent to the observational design, that should be taken into consideration when interpreting the findings. While our register-based design offers large sample sizes and real-world data, the study was limited by the variables available in the registers, thus introducing the possibility of confounding bias. Our data did not include the clinical indication, dose, or duration of use of the prescribed drugs. Side effects of antipsychotics may be dose- and duration-dependent [12], which highlights a limitation of our dataset and potentially restricts the conclusions that can be drawn from the results. The prescription register does not cover drugs used in hospital wards or other inpatient settings. As a register study, we did not have access to individual clinical patient data or information on risk factors such as alcohol use. While data on alcohol use before and after diagnosis would have provided valuable insights, obtaining such data poses challenges. Limited studies conducted in non-institutionalized settings have suggested that alcohol misuse continues among patients with alcohol-related neurocognitive disorders after diagnosis. For example, Sanvisens et al. [32] found that two-thirds of WKS patients continued to consume alcohol after hospital discharge, and our own previous findings demonstrated that WKS and ARD patients experience increased mortality due to alcohol-related causes [25]. While diagnoses of WKS and ARD were made by clinicians during standard course of care and could not be independently verified, dementia diagnoses in Finnish healthcare registers in general have very good accuracy [33]. Earlier studies have shown that WKS and ARD are underdiagnosed conditions [34,35], which implies that the diagnoses may be skewed towards more severe cases.

## 7. Implications and Conclusions

The present study has several clinical implications. Our findings raise the issue of possible overuse of antipsychotic medication in patients diagnosed with WKS or ARD. Current recommendations suggest that antipsychotics for NPS in dementia patients be avoided unless nonpharmacologic options have failed [36,37]. However, our data suggest that antipsychotic drugs may not increase mortality in this patient group, thus raising the question of whether different dementia disorders need tailored recommendations for treatment of NPS.

It is crucial to consider all strategies for reducing alcohol consumption in patients with alcohol-related neurocognitive disorders. Our findings suggest that antipsychotics might have potential utility in this regard, although additional research is required. Use of antipsychotics in progressive dementia disorders is associated with increased mortality. Antipsychotics also have other severe side effects, including adverse metabolic effects and extrapyramidal symptoms, which necessitate caution in their use across all patient groups. However, in patients with alcohol-related neurocognitive disorders, this study suggests that the increase in mortality may not be a significant reason to avoid the use of antipsychotics. Exploring newer antipsychotic medications with fewer metabolic and cardiovascular side effects, such as dopamine partial agonists, could prove beneficial and warrants further investigation in this patient population.

It is important to note that our findings are preliminary and do not imply a protective effect of antipsychotics in patients with alcohol-related neurocognitive disorders. Future research is warranted to quantify the effect of antipsychotics on mortality and other relevant outcomes such as severity of NPS and levels of alcohol consumption. Steps should be taken to improve access to healthcare and alcohol treatment services for these patients.

In conclusion, this is the first population-based study to examine antipsychotic use and its effect on mortality among patients with alcohol-related neurocognitive disorders. The major findings to emerge were the high prevalence of antipsychotic use and the lower mortality among antipsychotic users than among non-users in those diagnosed with WKS or ARD. These results are in contrast to earlier studies in which the use of antipsychotics has been associated with an elevated mortality risk in older people and persons with dementia. Unlike in progressive dementia disorders, it is possible that the use of antipsychotic medication among persons with WKS or ARD affects their overall situation in a way that results in decreased all-cause and alcohol-related mortality.

## Figures and Tables

**Table 1:** Characteristics of the study population.

	Antipsychotic Medication Use		*p*-Value
Characteristics by Diagnosis Group	Non-User	User	
WKS (N = 1149)			
Number (%)	736 (64.1)	413 (35.9)	
Men, n (%)	544 (73.9)	297 (71.9)	0.46
Person-years followed up	5769	3421	
Number of deaths	428	217	
Mean age at diagnosis, mean (SD)	57 (9)	57 (9)	0.96
Crude mortality *, % (95% CI)	68 (64–72)	64 (58–71)	0.044
Standardized mortality ratio (95% CI)	5.95 (5.41–6.54)	5.20 (4.55–5.94)	0.11
ARD (N = 2432)			
Number (%)	1497 (61.5)	935 (38.5)	
Men, n (%)	1166 (77.9)	726 (77.6)	0.88
Person-years followed up	7167	5453	
Number of deaths	984	507	
Mean age at diagnosis, mean (SD)	66 (10)	64 (9)	<0.001
Crude mortality *, % (95% CI)	86 (82–89)	77 (71–82)	<0.001
Standardized mortality ratio (95% CI)	5.87 (5.52–6.25)	4.70 (4.31–5.13)	<0.001

* Cumulative mortality rate at 15 years. ARD: alcohol-related dementia; WKS: Wernicke–Korsakoff syndrome.

## Data Availability

Researchers who wish to replicate our study may apply for permission to use the registers of the Finnish Institute for Health and Welfare (info@thl.fi) for data from the Care Register for Health Care; Kela (tietoaineistot@kela.fi) for data on medication purchases; and Statistics Finland (info@stat.fi) for data on population, mortality, and causes of death. 31 December 2018 (for register data) and 31 December 2020 (for additional mortality data).
